# Metabolic Reconstruction and Modeling Microbial Electrosynthesis

**DOI:** 10.1038/s41598-017-08877-z

**Published:** 2017-08-21

**Authors:** Christopher W. Marshall, Daniel E. Ross, Kim M. Handley, Pamela B. Weisenhorn, Janaka N. Edirisinghe, Christopher S. Henry, Jack A. Gilbert, Harold D. May, R. Sean Norman

**Affiliations:** 1 Biosciences Division, Argonne National Laboratory, Argonne, Illinois USA; 2 Department of Ecology and Evolution, The University of Chicago, Chicago, Illinois USA; 3 Department of Surgery, The University of Chicago, Chicago, Illinois USA; 4 Computing, Environment, and Life Sciences, Argonne National Laboratory, Argonne, Illinois USA; 5 Department of Microbiology and Immunology, Medical University of South Carolina, Charleston, South Carolina USA; 6 Marine Biomedicine and Environmental Science Center, Medical University of South Carolina, Charleston, South Carolina USA; 7 Department of Environmental Health Sciences, Arnold School of Public Health, University of South Carolina, Columbia, South Carolina USA; 8 Present Address: Department of Microbiology and Molecular Genetics, University of Pittsburgh, Pittsburgh, Pennsylvania United States; 9 Present Address: School of Biological Sciences, The University of Auckland, Auckland, New Zealand

## Abstract

Microbial electrosynthesis is a renewable energy and chemical production platform that relies on microbial cells to capture electrons from a cathode and fix carbon. Yet despite the promise of this technology, the metabolic capacity of the microbes that inhabit the electrode surface and catalyze electron transfer in these systems remains largely unknown. We assembled thirteen draft genomes from a microbial electrosynthesis system producing primarily acetate from carbon dioxide, and their transcriptional activity was mapped to genomes from cells on the electrode surface and in the supernatant. This allowed us to create a metabolic model of the predominant community members belonging to *Acetobacterium*, *Sulfurospirillum*, and *Desulfovibrio*. According to the model, the *Acetobacterium* was the primary carbon fixer, and a keystone member of the community. Transcripts of soluble hydrogenases and ferredoxins from *Acetobacterium* and hydrogenases, formate dehydrogenase, and cytochromes of *Desulfovibrio* were found in high abundance near the electrode surface. Cytochrome c oxidases of facultative members of the community were highly expressed in the supernatant despite completely sealed reactors and constant flushing with anaerobic gases. These molecular discoveries and metabolic modeling now serve as a foundation for future examination and development of electrosynthetic microbial communities.

## Introduction

A microbial electrosynthesis system (MES) is a bioelectrochemical device that employs microbes to generate, or synthesize, valuable products from CO_2_ and electrons from the cathode^1^. This technology has potential for the renewable generation of biofuels and commodity chemicals, therefore understanding which microbes and which metabolic pathways are involved on biocathodes is critical to improving the performance of MESs. Furthermore, this work has broader environmental implications including understanding ecological aspects of one carbon metabolism and extracellular electron transfer relevant to global biogeochemical cycling.

Advances in microbial electrosynthesis and related biocathode-driven processes have primarily focused on the production of three compounds: hydrogen^2^, methane^3^, and acetate^4^. However, microbial electrosynthesis can be used to generate value-added multi-carbon compounds such as alcohols and various short-chain fatty acids^5^. To manipulate (and optimize) these systems it is valuable to understand the metabolic pathways and extracellular electron transfer (EET) enzymes or molecules involved in cathode oxidation, and we must determine how these cathodic electron transport components are coupled to energy conservation by the microbial cell. While an increasingly robust body of literature has been established relating to microbial communities metabolizing with an anodic electron acceptor^6–8^, much is still unclear about the diverse metabolic capabilities of microorganisms and communities growing on a cathode^9^. Recently, a multi-omics evaluation of a mixed-species, carbon dioxide-fixing aerobic biocathode poised at +310 mV versus a standard hydrogen electrode (SHE) revealed that an uncharacterized *Chromatiaceae* was the most likely candidate for CO_2_ fixation coupled to biocathode growth^10^. The primary carbon fixing and hypothetical electron transport pathways associated with that microbial electrosynthesis system were described^10^, illustrating the value of the multi-omics approach to understand complex electrode-associated communities.

In our previous work, we discovered a mixed microbial community that produced primarily acetate and hydrogen from carbon dioxide with cathodes poised at −590 mV versus SHE^11^. Long-term operation of these mixed species MESs yielded some of the highest rates reported for microbial electrosynthesis^12, 13^. However, little is known about the microbial taxa and metabolism responsible for cathode-driven autotrophy at low electrode potentials. To address some of these knowledge gaps about metabolic diversity in relation to microbial electrosynthesis, we used metagenomics, metatranscriptomics, and metabolic flux modeling to hypothesize carbon flux and EET mechanisms in a multispecies electrosynthetic microbial community. We have inferred interactions between diverse members of the microbial community, including interspecies metabolite transfer and oxygen scavenging, leading to a hypothetical multispecies biofilm model.

## Results and Discussion

### Performance and compositions of microbial electrosynthesis systems

**Figure 1:**
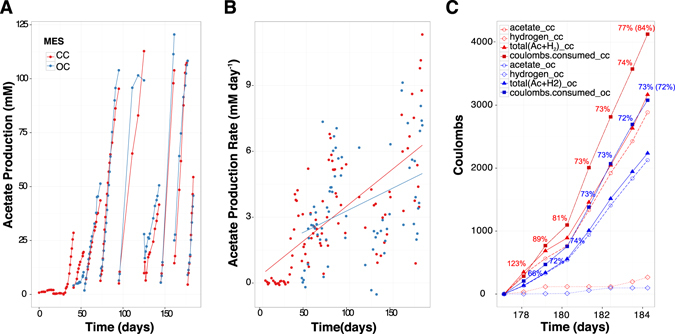
Reactor performance over time. (**A**) Acetate production in CC and OC – breaks in plotted lines indicate an exchange of the medium, (**B**) acetate production rate in CC and OC, and (**C**) accounting of the coulombs over a seven day period between medium exchanges in CC and OC, numbers are percent coulombic efficiency with total percent efficiency averaged over the 7 days in parentheses.

Two of the five previously described MESs^12^ were selected for multi-omic analyses in order to determine microbial metabolic capability and metabolism linked to cathodic electron transfer. Both reactors were inoculated from an established electrosynthetic community poised at −590 mV vs. a standard hydrogen electrode (SHE), and operated for over 100 days. The two reactors generated on average 65% acetate, 34% hydrogen, 0.4% formate, 0.3% propionate and 0.2% butyrate from CO_2_ and the electrode (Fig. 1). The production rates and titers far exceeded what could be explained by the very small amounts of abiotic hydrogen production (45 μM day^−1^) observed in identical abiotic reactors^11^. Furthermore, cyclic voltammetry scans comparing electrode-attached microbes to blank or filtered supernatant scans clearly demonstrated microbial-dependent electron transfer at −590 mV^12^.

**Figure 2:**
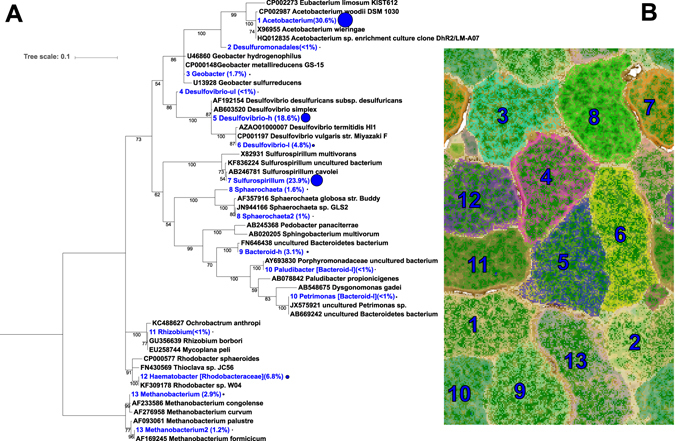
(**A**) Phylogenetic tree of the CCc microbial community using EMIRGE-based reconstructed 16S rRNA gene sequences (blue) and relative abundance values in parentheses and indicated by the relative size of the blue circle. Sequences were aligned using MUSCLE and the evolutionary history was inferred using the Maximum Likelihood method based on the Jukes-Cantor model. Bootstrapping support greater than 50% is indicated on the tree and is based on 1,000 iterations. (**B**) ESOM based on tetranucleotide frequency in the CC cathode metagenome.

At the conclusion of the experiment, one of the two reactors was left at open circuit for three hours to distinguish between transcripts influenced by the supply of electrons from the cathode compared to electrons supplied by hydrogen and/or other free metabolites in the biofilm. Similarly, metatranscriptome samples were taken from the electrode surface and compared to the supernatant where hydrogen was routinely measured. This led to four metatranscriptome samples from two reactors: closed circuit cathode (CCc), closed circuit supernatant (CCs), open circuit cathode (OCc), and open circuit supernatant (OCs). The closed circuit reactor was operated for 184 days prior to harvesting and the open circuit reactor was inoculated from CC after 43 days of initial operation and then both were treated with identical sampling and medium exchange regimes. Coulombic efficiencies at time of sampling (before switching OC reactor to open circuit) were 77% and 73% from CC and OC, respectively (Fig. 1c). Thirteen genome bins spanning 5 different phyla were recovered from the assembled metagenomes taken from the closed circuit MES (Fig. 2). Near-complete genomes (89–100% complete) were produced for *Sulfurospirillum* str. MES7, *Acetobacterium* str. MES1, *Desulfovibrio* str. MES5, *Methanobacterium* str. MES13, *Bacteroides* str. MES9, *Geobacter* str. MES3, and *Sphaerochaeta* str. MES8 (Supplementary Table 2), and a second partially complete (~65%) *Desulfovibrio* str. MES6 genome was further obtained. *Acetobacterium*, *Sulfurospirillum*, and *Desulfovibrio* combined comprised 40–90% of the community in each condition, with a Rhodobacteraceae related organism also consistently represented (4–20% relative abundance) (relative abundance of each organism in CCc can be found in Fig. 2 and relative abundance in all conditions, including 3 additional reactors can be found in Supplementary Figure 2). The 13 annotated genome bins recovered from the CC reactor were used as the basis for transcript mapping and analyses. The following analyses describe pathways discovered in the metagenomes, their importance supported by metabolic models, and how the models and transcriptomes indicate flux through those pathways. The transcript abundance and flux balance analysis provide important, but preliminary insights into biocathode-associated metabolism.

### Description of the microbial communities

Three species, *Acetobacterium* (designated here as str. MES1), *Sulfurospirillum* str. MES7, and *Desulfovibrio* str. MES5, comprised ~72% relative abundance of the total community on the active cathode (CCc) and ~80% in the supernatant (CCs) (Supplementary Figure 2). The abundance, gene expression levels, and predominant activity in the reactor (acetogenesis) as well as the known physiology of similar organisms indicate that the *Acetobacterium* species is the principal carbon fixer on the electrode and thus the keystone species in the microbial community. The *Sulfurospirillum* is the predominant taxa in the supernatant in nearly every replicate reactor (Supplementary Figure 2) and has diverse metabolic capabilities. The gene expression profile of *Sulfurospirillum* points to a microaerophilic lifestyle. The *Desulfovibrio* spp. present in the reactors have a high number of transcripts mapping to genes encoding hydrogenases, formate dehydrogenase, and cytochromes on the electrode and are hypothesized to reduce protons to hydrogen at the electrode surface.

**Figure 3:**
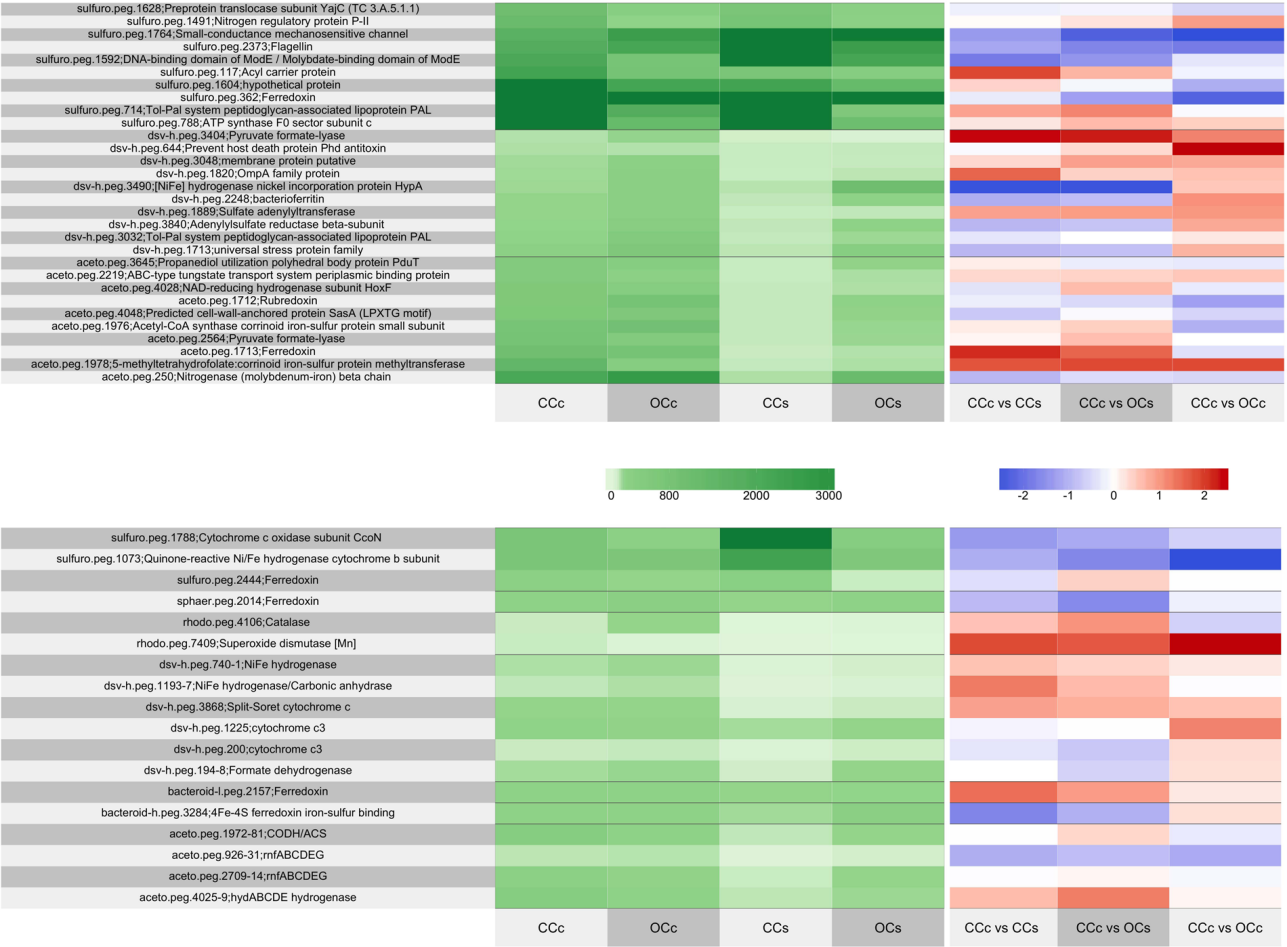
Expression profile and comparative expression of (**A**) the top 10 metabolic-related genes in the top 3 genome bins in the CCc reactor and (**B**) selected genes hypothesized to be important for cathode-associated growth. These genes were selected based on high differential expression between conditions instead of highest overall transcripts. The green panel shows relative expression between microorganisms and the blue and red panel compares expression between condition.

Genomic and transcriptomic evidence indicates the *Acetobacterium* str. MES1 reduces CO_2_ to acetate through the Wood-Ljungdahl pathway (WLP). WLP-indicative acetyl-CoA synthase/carbon monoxide dehydrogenase (ACS/CODH, aceto.peg.1971–1981) and 5-methyltetrahydrofolate:corrinoid iron-sulfur protein methyltransferase (aceto.peg.1978) components were among the most highly expressed genes on the closed circuit electrode surface (Fig. 3). Furthermore, the canonical enzyme of the WLP, ACS/CODH, had on average >3-fold greater expression on the closed-circuit electrode compared to the supernatant and higher expression on CCc compared to OCc. Interestingly, a *Clostridium*-type chain elongation pathway^14^ was expressed in the *Acetobacterium* str. MES1 to convert acetyl-CoA to butyrate (aceto.peg.1850-4, 2312), which may explain the butyrate production by the electrosynthetic microbiome (Supplementary Figure 3)^12^.

A soluble uptake hydrogenase complex, *hydABCDE* (aceto.peg.4025-9), was the most highly expressed *Acetobacterium* str. MES1-associated hydrogenase genes on the electrode. This hydrogenase complex is homologous to the *A*. *woodii* HydABCD hydrogenase complex and is therefore likely to provide *Acetobacterium* str. MES1 with reducing equivalents (ferredoxin and NADH) for carbon dioxide fixation through the Wood-Ljungdahl pathway. The high expression of *hydABCDE* and the *rnfABCDEG* gene clusters, suggests *Acetobacterium* str. MES1 conserves energy by generating a sodium-dependent transmembrane electrochemical gradient similar to *A*. *woodii* ^15^.

A key difference between strain MES1 and *A*. *woodii* is that strain MES1 has two Rnf complex copies, one with 15-fold greater gene expression on the electrode surface (high: aceto.peg.2709–14 and low: aceto.peg.926–931). While it is unknown whether Rnf participates in extracellular electron transfer, it is essential for autotrophic growth and Kracke *et al*. proposed the complex as a possible conduit to the electrode^16^. The multiple copies with differential expression is similar to *Azotobacter vinelandii*, whose copy expression disparity is driven by ammonium depletion^17^. Rnf expression positively correlates with nitrogen fixation (*nif*) gene expression because Rnf is required for stable accumulation of the 4Fe4S clusters in *nifH* ^17, 18^. *Acetobacterium* str. MES1 also maintains a nitrogenase gene cluster (*nifBEHN*) (aceto.peg.248–54) with an adjacent ferredoxin. In fact *nifH* was in the top 5 most expressed *Acetobacterium* str. MES1 genes in all conditions, suggesting N-limitation and/or an involvement in electron transfer (Fig. 3a). As ammonium is depleted, nitrogenase has been shown to reduce protons to hydrogen in the absence of N_2_ as a substrate^19^. This may lead to hydrogenesis by *Acetobacterium* if excess reducing potential is applied (e.g. by the electrode) and ammonium is low. This supports previous observations of a lag time between fresh medium exchange and the onset of hydrogenesis where ammonium would be depleted after a few days, and of a positive correlation between *Acetobacterium* abundance and hydrogen production^13^. It is also possible that electron transfer from the Rnf complex to the nitrogenase for proton reduction is a source of hydrogen production by the electrosynthetic microbiome in this and related studies^13^, and is a potential target for biotechnological exploitation^19^.

*Sulfurospirillum* str. MES7 remained consistently abundant (>60% relative abundance) in the supernatant over the duration of the experiment despite frequent exchanges with fresh medium^11, 12^. However, the role of *Sulfurospirillum* in electrosynthesis has remained elusive. Given its prevalence it must fix CO_2_ and/or utilize acetate as a carbon source. In support of CO_2_ fixation, *Sulfurospirillum* spp. are hypothesized to fix CO_2_^20^ and other members of the epsilonproteobacteria can fix CO_2_ via the reductive tricarboxylic acid (rTCA) cycle^21^. As shown here and a recent *Sulfurospirillum* comparative genomic study^22^, the *Sulfurospirillum* str. MES7 assembly contains several genes necessary to overcome the irreversible enzymes of the TCA cycle, including a 2-oxoglutarate oxidoreductase alpha, beta, delta, and gamma subunits (EC 1.2.7.3, sulfuro.peg.1237–40) and fumarate reductase (EC 1.3.5.1, sulfuro.peg.592-4). No ATP citrate lyase or citryl-CoA lyase were found, but it does have genes for the conversion of citrate to oxaloacetate + acetate (citrate lyase alpha and beta chains, and citrate (pro-3S)-lyase, EC 4.1.3.6, sulfuro.peg.2133-6), suggesting the possibility of CO_2_ fixation through rTCA. This is also supported by the high transcriptional activity of the key irreversible genes in the rTCA cycle (Supplementary Figure 4). In addition, acetate permease (sulfuro.peg.1580) and acetate kinase (sulfuro.peg.1274) are both highly expressed, indicating either that a supplementary organic carbon source might be required for growth, or that *Sulfurospirillum* str. MES7 flexibly switches between CO_2_ fixation and acetate oxidation in the MES reactor.

By far the most highly expressed genes relating to terminal electron accepting processes in *Sulfurospirillum* str. MES7 (for any condition) were cytochromes, particularly cytochrome c oxidases. C-type heme-copper oxidases are the final step in the catalytic reduction of oxygen in certain bacteria^23^ and *ccoNOPQ* is highly expressed by *Sulfurospirillum* str. MES7 (sulfuro.peg.1785-8) in all MES conditions. The high expression of *ccoNOPQ* suggests *Sulfurospirillum* str. MES7 is growing microaerobically in the MES, using oxygen as the terminal electron acceptor. This supports the hypothesis that microaerophilic bacteria provide a supportive role to the rest of the community by scrubbing low levels of oxygen that diffuse from the anode chamber. These potentially parasitic reactions might be avoided by constantly flushing the anode chamber with anoxic gases to eliminate oxygen diffusion.

*Desulfovibrio* str. MES5 was the 3^rd^ most abundant taxon on the electrode surface. Based on transcription patterns, we hypothesize that *Desulfovibrio* converts CO_2_ to formate while using cytochromes, formate dehydrogenase, and/or hydrogenases to accept electrons from the cathode. The high expression of formate dehydrogenase (3-fold, dsv-h.peg.194) and multiheme cytochromes (6-fold, dsv-h.peg.1195; 6-fold, dsv-h.peg.3868) on the electrode compared to the supernatant supports this hypothesis. We further propose that *Desulfovibrio* str. MES5 reduces protons as a terminal electron acceptor and generates hydrogen gas^24^, which is supported by the comparatively high expression of hydrogenases (dsv-h.peg.740-1) on the electrode surface (>3-fold CCc vs. CCs). In further support of this, *Desulfovibrio* spp. have been shown to generate hydrogen from low potential redox donors^25^ as well as interact with a cathode in a similar manner to generate hydrogen via electrohydrogenesis^26–28^. It is likely that acetate from *Acetobacterium* str. MES1 is used as a supplementary carbon source for *Desulfovibrio* str. MES5^29^, but based on the high hydrogenase relative to the low acetate kinase expression by *Desulfovibrio* str. MES5, we hypothesize that the majority of reducing potential comes from the electrode. It is possible that the 2-bromoethanesulfonate (2-BES) occasionally added to suppress methanogenesis could be degraded and the sulfonate moiety reduced to sulfide by *Desulfovibrio*; indeed dissimilatory sulfite reductase genes were highly expressed, but analysis of closely related strains suggest *Desulfovibrio* lack this capacity^30^. Biodegradation of 2-BES to sulfide and acetate would not result in cathode-dependent metabolism, but it is well established that *Desulfovibrio* will interact with a cathode and produce H_2_^2, 26–28^. Therefore the discussion and the model are focused on the latter.

While *Acetobacterium* str. MES1, *Sulfurospirillum* str. MES7, and *Desulfovibrio* str. MES5 can explain most of the activity occurring in the microbial electrosynthesis systems, the remaining genomes have broad metabolic capabilities, but are typically in lower abundance (<7%). We hypothesize that they are consuming detritus, degradation products, hydrogen, and/or short chain fatty acids generated by the abundant organisms. A pan-genome associated with the Rhodobacteraceae family had an expression profile consistent with acetate oxidation, hydrogen consumption, and *cbb3*-type cytochrome c oxidases that could be indicative of microaerobic growth. An uptake hydrogenase (rhodo.peg.11021) and cytochrome c oxidase (rhodo.peg.215) had higher expression in both supernatants compared to both electrodes. Cytochrome c oxidases have been expressed in *Rhodobacter* sp. growing in microaerobic and anaerobic conditions^31^ and were also found to be upregulated by *Shewanella* *oneidensis* MR-1 growing on electrodes^32^. While we are hypothesizing that these cytochrome c oxidases in Rhodobacteraceae str. MES12 and *Sulfurospirillum* str. MES7 are involved in microaerobic growth, given their prominence in this study and others involving bioelectrochemical systems^32^, their role in EET should be investigated further. One of two *Bacteroides* genomes (str. MES10) was closely related (93% sequence identity) to *Proteiniphilum acetatigenes*, which has been shown to generate acetate when growing on wastewater cell debris^33^ and Croese *et al*. discovered a relatively high abundance of uncultured *Bacteroides* in a hydrogen producing biocathode community^34^, suggesting possible productive roles for the *Bacteroides* str. MES9 and MES10 in the electrosynthetic microbiome. Interestingly, *Bacteroides* str. MES9 expresses a butanol dehydrogenase (bacteroid-h.peg.1751) on the electrode surface, which suggests that changing the operating conditions of the MES could produce biofuels and other valuable products (see Bioprospecting section in supporting materials). Additionally, both *Bacteroides* genomes and the *Sphaerochaeta* str. MES8 genome exhibited high expression of ferredoxin genes (bacteroid-h.peg.3284, bacteroid-l.peg.2157, sphaer.peg.2014), which may be used to shuttle electrons for community metabolism.

### Redox-active components of the biocathode community

Generally, the proposed mechanisms for cathodic electron transfer in the absence of added mediators are analogous to anodic electron transfer, namely that cytochromes are the primary conduit of electrons to and from the microbial cell^35–39^. The role of cytochromes on anodes has been primarily elucidated in pure culture, but metatranscriptomic studies also point to cytochrome involvement in EET to anodes containing mixed microbial communities^6, 7^. Much less is known about EET in communities grown on cathodes. A putative periplasmic cytochrome was essential for cathodic current in fumarate reducing biocathodes^40^ and cytochromes were also proposed to mediate EET at an oxygen-reducing biocathode^10^. In this study, *Desulfovibrio* str. MES5 has three highly expressed, multiheme cytochromes containing signal peptides on the electrode: two with 6 times higher (dsv-h.peg.1195 and dsv-h.peg.3868) and one with a 2.5 times higher expression (dsv-h.peg.1225) on the electrode compared to the supernatant. The latter two had greater expression (2x) on the closed-circuit cathode compared to the open circuit cathode. Additionally, a putative periplasmic formate dehydrogenase from *Desulfovibrio* str. MES5 (dsv-h.peg.194) was expressed nearly 3 times higher on the electrode compared to supernatant, >2x CCc versus OCc, and could explain formate transiently observed in the supernatant (Supplementary Figure 3)^12, 41^. Further tests will be needed to validate the importance of these redox active enzymes on the electrode. In addition to their possible involvement in electron transfer at cathode surfaces, cytochromes can also act as natural mediators for hydrogenases^42, 43^ and could shuttle electrons from the electrode or from electrode-attached hydrogenases to the microbial community. Three NiFe hydrogenases were highly expressed by *Desulfovibrio* str. MES5 on the electrode compared to the supernatant (dsv-h.peg.740–741, >3-fold and 6-fold increase on electrode compared [CCc] to supernatant [CCs]; and dsv-h.peg.1193, 6-fold increase on electrode [CCc] compared to supernatant [CCs]). One of these hydrogenases (dsv-h.peg.740) was predicted to be secreted out of the cytoplasm and another (dsv-h.peg.1193) was adjacent to a high molecular weight cytochrome c (dsv-h.peg.1195) and predicted to have transmembrane domains. Given the relatively high expression on the cathode compared to supernatant, it is hypothesized that cytochromes, hydrogenases, and possibly formate dehydrogenases associated with *Desulfovibrio* str. MES5 act as electron mediators between the electrode and the cell to deliver reducing equivalents. In addition to the well-established role of cell-bound cytochromes in direct electron transport with an electrode^44, 45^, free cytochromes have been shown to be integral to anodic electron transfer while still imbedded in the biofilm matrix, and could be assisting electron transport to the community in this electrosynthetic biofilm^38^.

The recent discovery that extracellular enzymes (likely hydrogenases and formate dehydrogenases) may be present in cathodic biofilms and may catalyze electron transfer into microbially-utilizable substrates (hydrogen and formate) adds a new and important piece to solve the EET mechanistic puzzle^46^. Of the genes highly expressed on the electrode, several redox-active electron carriers (particularly hydrogenases and ferredoxins) had high expression on the poised electrode compared to the supernatant and open circuit electrode. A *Sulfurospirillum* str. MES7 ferredoxin (sulfuro.peg.2444) is >2-fold higher on the electrode compared to the supernatant (1.5x higher CCc vs. OCc) and the low abundance *Bacteroides* str. MES10 contains a ferredoxin (bacteroid-l.peg.2157) that is >9-fold higher expression on the electrode compared to supernatant (1.7x higher CCc vs. OCc). Importantly, a ferredoxin from *Acetobacterium* str. MES1 (aceto.peg.1713) was 7-fold higher on the electrode (CCc) compared to both supernatant conditions (CCs and OCs). Additionally, the highly expressed *Acetobacterium* str. MES1 hydrogenase gene cluster, *hydABCDE* (aceto.peg.4025–4029), had >5-fold higher expression per cell on the electrode compared to the supernatant (1.6x higher CCc vs. OCc), despite the presence of hydrogen in the supernatant. The combination of soluble hydrogenases and ferredoxins on the cathode could facilitate cathodic electron transfer into organisms like *Acetobacterium* that contain no cytochromes and no obvious means of electron transfer through the cell envelope.

Despite the lack of a redox-active signal in cell-free cyclic voltammograms and a catalytic wave characteristic of direct electron transfer in the electrosynthetic microbial community^12^, SEM-EDX images show extracellular material with elemental signatures of common redox-active enzymes (Supplementary Figure 5). Additionally, no reduction in current or shift in the voltammetric peaks were observed when the supernatant was replaced with fresh medium, suggesting tightly bound electron transfer mechanisms. The discovery of metals such as iron and nickel on the electrode surface could be concentrated enzymes, but microbial or pH induced precipitation of the metals as (hydr)oxides, sulfides, carbonates, phosphates, perhaps even as nanoparticles cannot be ruled out. The latter has been hypothesized by Jourdin *et al*. who observed copper concentrated on the surface of a microbial electroacetogenic cathode^47^. Whether precipitated metals, extracellular enzymes, or cell envelope-bound components, it is clear that the redox active components and electrochemical activity are inseparable from activity by the biofilm community.

Based on the highly expressed redox active proteins on the electrode (all of the proteins mentioned above are in the top 5% of total genes expressed on the electrode), and the consistent hydrogen production in the reactors^11–13^, extracellular enzymes and/or concentrated metals are likely functionalizing the electrode while hydrogen, ferredoxins, and/or cytochromes act as shuttles for different organisms in the biofilm. This would explain why microbes lacking an outer membrane can thrive on an electrode, and why previous studies ran electrohydrogenic biocathodes that could sustain hydrogen generation in the absence of a carbon source for over 1000 hours^2^. Further studies are needed to fully characterize functionalization of electrodes by microbes to optimize this strategy.

### Metabolic model reconstructions and analysis

Genome-scale metabolic models were constructed for the three predominant taxa in the electrosynthetic community (see methods): *Acetobacterium* str. MES1, *Sulfurospirillum* str. MES7, and *Desulfovibrio* str. MES5. These models were then used, in combination with flux balance analysis^48^, to predict the metabolic activity of each of these species during microbial community growth on the electrode. The accuracy of these flux predictions was evaluated by calculating the fraction of active reactions predicted by the models that were associated with actively expressed genes, as determined from the metatranscriptomic data (Table 1 and methods). In this analysis, the *Acetobacterium* model displayed only one plausible flux profile, which involved carbon fixation and acetate production via the Wood-Ljungdahl pathway using hydrogen, electrons, or similar reducing equivalents as an electron source. With this flux profile, there were 338 active reactions with associated genes in *Acetobacterium*, and for 258 (76%) of these reactions, at least one of the associated genes was actively expressed. Most active reactions that lacked expression support were involved in either amino acid biosynthesis or nucleotide metabolisms.

**Table 1:** Transcriptome support for metabolic activity predicted by models.

Species	Description	Active reactions^^^	Supported reactions*	Expressed genes
*Acetobacterium*	CO_2_ fixation to acetate	338/1244	258/338	402/954
*Sulfurospirillum*	Reduction of CO_2_	328/1143	219/328	252/661
*Sulfurospirillum*	Oxidation of acetate	330/1142	218/330	252/661
*Desulfovibrio*	Conversion of CO_2_ to formate	322/1124	244/322	425/759
*Desulfovibrio*	Consumption of acetate	320/1124	243/320	425/759

^^^Active reactions only include reactions with associated genes (gapfilled reactions filtered out). *Supported reactions are active reactions (gapfilled reactions filtered out) associated with at least one actively expressed gene.

The *Sulfurospirillum* model predicted two alternative theories for the metabolic role of this species: (i) reduction of CO_2_ through the reductive TCA cycle with hydrogen used as the reducing agent (67% of active reactions associated with at least one expressed gene), and (ii) oxidation of acetate coupled to O_2_ reduction (66% of active reactions associated with at least one expressed gene). Given the nearly equal agreement of both of these operating with our transcriptome data, and given the high abundance of *Sulfurospirillum* in our community, it is possible *Sulfurospirillum* actually performs both roles depending on its context and environment.

The *Desulfovibrio* model also predicted two alternative theories for the metabolic role of this species in our electrosynthetic microbiome: (i) conversion of CO_2_ and electrons/hydrogen to formate (75.7% of active reactions associated with at least one expressed gene), or (ii) consumption of acetate (75.9% of active reactions associated with at least one expressed gene). As with *Sulfurospirillum*, the expression data was equally supportive of both metabolic theories, indicating that *Desulfovibrio* also performs a combination of carbon-fixation and acetate utilization.

**Figure 4:**
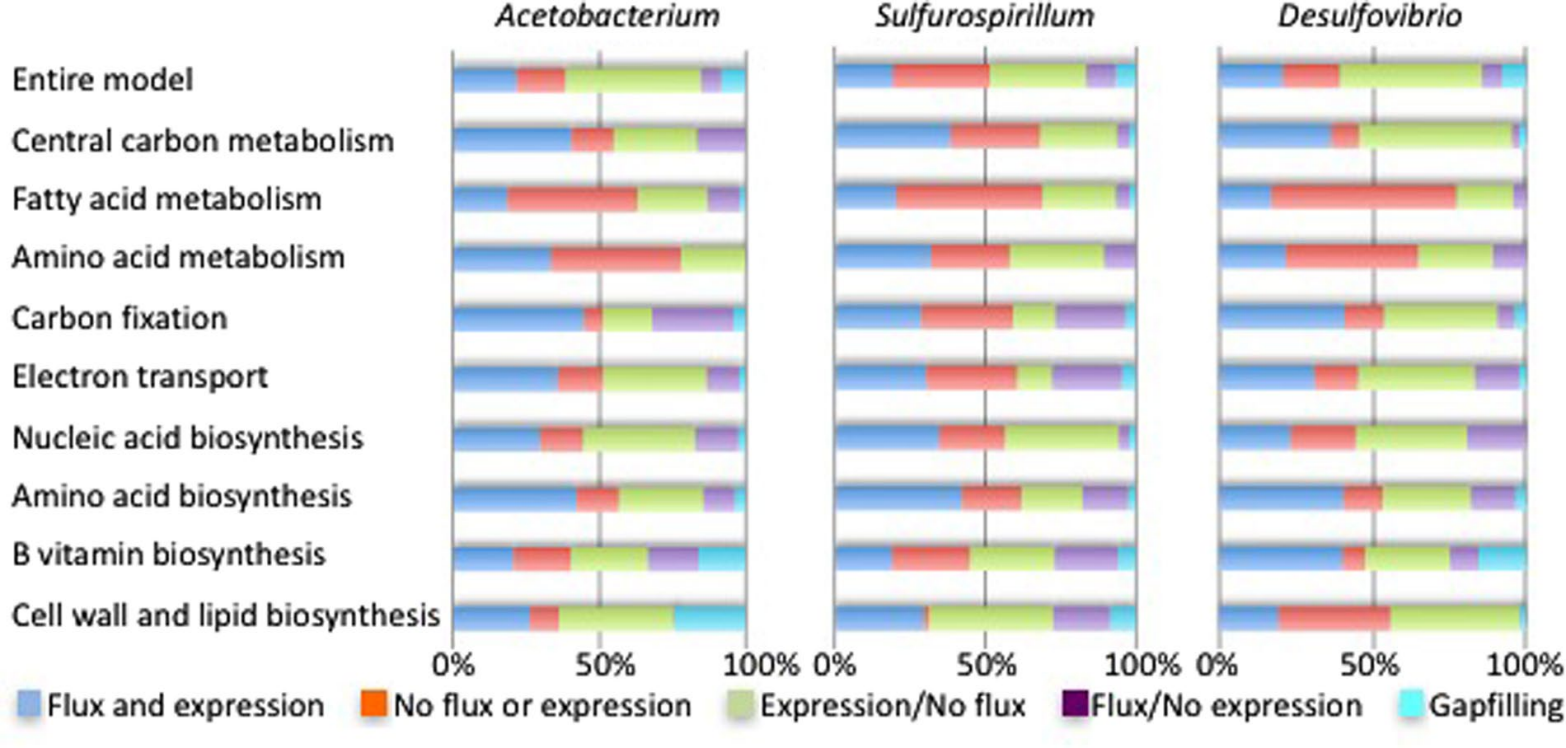
Pathway flux and model agreement with expression data. The degree of agreement between the model-based flux predictions and expression data for each of the three metabolic models is shown, both for the entire models and broken down by categories of metabolism. In the graph, reactions are divided into five categories based on their flux and the expression of their associated genes: (i) reactions that are active and associated with at least one expressed gene (dark blue); (ii) reactions that are inactive and associated only with unexpressed genes (dark red); (iii) reactions that are inactive and associated with one or more expressed genes (green); (iv) reactions that are active and associated only with unexpressed genes (purple); and (v) gapfilled reactions associated with no genes. The dark blue and dark red categories indicate agreement between the models and expression data; purple and green categories indicate disagreement.

In addition to evaluating the overall evidence supporting each of our predicted flux profiles, the transcriptome data was also applied to evaluate the flux profiles on a pathway-by-pathway basis, enabling a better understanding of model accuracy, and revealing insights into potential interspecies interactions (see Methods and Fig. 4). Generally, this analysis showed a large degree of agreement between predicted flux profiles and expression data, with some notable exceptions: (i) the terpenoid pathways in all our models were gap-filled because the models all include ubiquinone as a component of biomass, but there is little evidence for these pathways in our genomes and it is likely these genomes are functioning anaerobically and do not have to produce ubiquinone; (ii) the *Acetobacterium* and *Desulfovibrio* models both appear to overuse their pentose-phosphate-pathways compared to what would be expected based on expression data; (iii) the *Desulfovibrio* model overuses its thiamin pathway and should actually obtain thiamin from an external source according to expression data; (iv) the *Sulfurospirillum* model overuses its sulfur and nitrogen metabolism pathways; and (v) all three models appear to underutilize the vitamin B6 and fructose and mannose metabolism pathways.

The pathway-based model analysis (Fig. 4, Supplementary Figure 6) reveals interactions and dependencies among the top genomes. *Desulfovibrio* appears to require an external source of histidine, thiamin, riboflavin, and folate, while the other genomes or growth medium could be sources of all four of these compounds. *Acetobacterium* appears to be unique in making glutathione, and also has far more representation in carbon fixation than the other genomes. In contrast, *Sulfurospirillum* appears unique in possessing an active glyoxylate metabolism and the most active TCA cycle. It is interesting to note that all three genomes appear to produce most of their own amino acids, vitamins, and nucleotides, with *Desulfovibrio* being the least prototrophic of the three. This characteristic may be an essential component to successful biocathode-associated microorganisms where nutrients can be quickly depleted or unavailable. Biochemical reaction equations, compounds, associated flux values, and comparative genomics tools including hypothetical gene knockout experiments for all the models can be found at the KBase Narrative Interface: https://narrative.kbase.us/narrative/ws.15248.obj.1.

**Figure 5:**
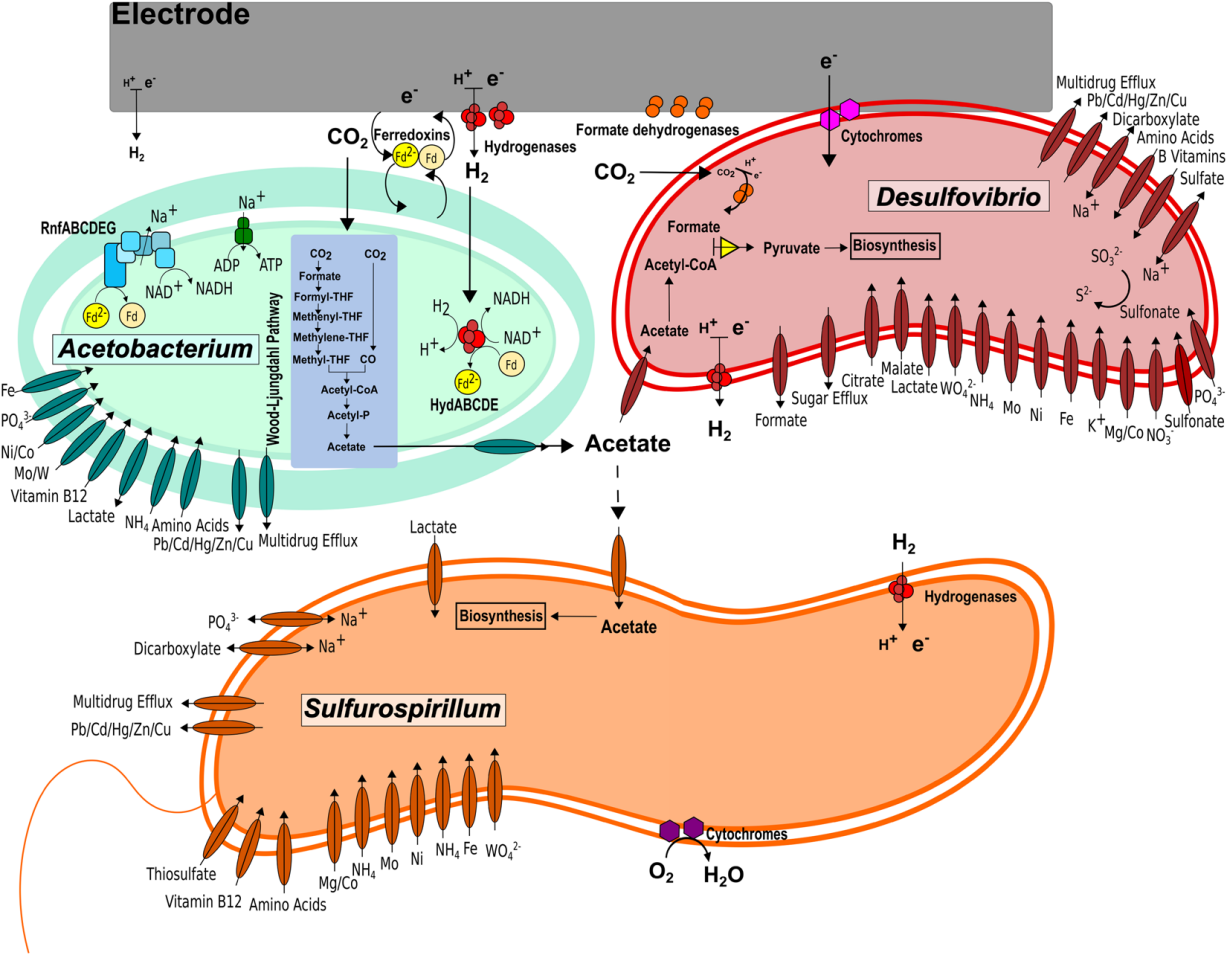
Hypothetical model of key metabolic activities and interactions among dominant members of the electrosynthetic microbial community.

Metabolic analysis and modeling of this unique microbial community capable of converting CO_2_ into volatile fatty acid provides a detailed look into its metabolism in a biocathode. The genome-wide metabolic capabilities of microorganisms in the community were determined, and a putative metabolic model of the primary community members has been summarized (Fig. 5). CO_2_ fixation and the carbon flux through the electrosynthetic microbial community center on the Wood-Ljungdahl pathway of the *Acetobacterium* genome, components of which were more active on the closed-circuit electrode compared to any other condition tested, demonstrating the importance of electrode-associated growth by *Acetobacterium*. Other microorganisms in the community are hypothesized to contribute to overall reactor and community fitness by scrubbing toxic compounds (e.g. oxygen) and providing nutrient exchange. Given the proper conditions and selective pressures, this electrosynthetic microbial community has a wide range of biotechnological potential, including the production of alcohols, 2,3-butanediol, and polyhydroxyalkonoates (see supplementary note on bioprospecting and Supplementary Table 4 for more detailed information).

The modeling results are largely consistent with the hypotheses proposed based on the metatranscriptomic data and demonstrate how metabolic models are useful for interpreting expression data in order to predict the role of individual species participating within a mixed microbial community. The model predictions: (i) confirmedthe role of *Acetobacterium* as the primary species utilizing electrons to reduce CO_2_; (ii) identified the more complex behavior of *Sulfurospirillum* as both reducing CO_2_ and utilizing acetate; and (iii) provided more depth and detail into our understanding of the heterotrophic growth of *Desulfovibrio*. These refined models should prove to be a resource for ongoing efforts to understand, and ultimately design electrosynthetic microbiomes.

## Conclusions

We have described the predominant genomic features of a high-performing microbial electrosynthesis system generating commodity chemicals from carbon dioxide. We used these metagenomes to construct a metabolic flux model of the most abundant members of the microbial community and transcript expression data were used to validate these models. From these analyses, we were able to generate hypotheses for the active metabolic pathways and electron flux routes through the community. The primary limitation to this approach was the analysis on four locations (CCc, CCs, OCc, OCs) from two reactors, but we think this study provides valuable insight into the metabolic capabilities of understudied cathode-associated microorganisms. Furthermore, the metagenome, model, and gene expression data provide an excellent hypothesis-generating tool and set the stage for future examinations. More work will need to be done to confirm the active pathways and critical enzymes related to cathodic growth and electron transport.

We demonstrated that a diverse set of microorganisms could be active in limited niche space with carbon dioxide as the only carbon source and the electrode as the only electron donor. We hypothesize that predominant members of the community could provide an ecosystem service by scrubbing oxygen, making this and similar electrosynthetic microbial communities valuable for practical application. If deployed in true carbon-capture situations such as power plants or industrial exhaust lines, oxygen scrubbing to protect the electrosynthetic anaerobes will be important. Future work is focused on determining the expression of specific genes and metabolic enzymes identified here to be important for electron flux through the biofilm.

## Materials and Methods

### Reactor design and operation

The characteristics of the reactors containing the microbial communities used for metagenomics and metatranscriptomics in this study were described in detail in two previous studies^11, 12^. Of the five MES reactors previously described, two reactors were selected for further analysis in this study. The first reactor, closed circuit (CC), was operated for 184 days before termination (MES-BW4 from previous manuscript is CC reactor here). The second reactor, open circuit (OC, MES 1a from Marshall *et al*.^12^), was inoculated from CC and operated in closed circuit mode for 141 days before it was left at open circuit for three hours prior to termination. The open circuit interval was done to compare the effects of closed versus open circuit biofilms. The initial source of microorganisms was from a brewery wastewater retention basin that was then subjected to sequential transfers of the graphite and supernatant in bioelectrochemical systems before inoculation into the two MESs described in this study. The brewery wastewater inoculum was allowed to establish on graphite granule cathodes and then repeatedly washed with fresh defined medium containing per liter: 2.5 g NaHCO_3_, 0.6 g NaH_2_PO_4_ * H_2_O, 0.25 g NH_4_Cl, 0.212 g MgCl_2_, 0.1 g KCl, 0.03 g CaCl_2_, 20 mL vitamin solution, and 20 mL mineral solution. Subsequent, sludge-free transfers were made into 150 mL volume cathode chambers of MESs containing 30 g of graphite granules, 75 mL of medium, 80% N_2_ and 20% CO_2_, and poised at −590 mV vs. standard hydrogen electrode (SHE) for the duration of the experiments unless otherwise noted. The medium during the initial startup period did not contain sodium 2-bromoethanesulfonate (2-BES), but 10 mM 2-BES was periodically used to inhibit methanogenesis and maintain a predominantly acetogenic culture later in the study, including at the time of sampling for metagenome and metatranscriptome analyses (day 184). The headspace atmosphere was switched to constant flushing of 100% CO_2_ on day 75. Upon termination of the experiment, supernatant and bulk granular cathodes were removed for DNA and RNA extraction. All samples were frozen in liquid nitrogen within 5 minutes of applied voltage interruption, except OC which was deliberately left at open circuit for 3 hours prior to freezing. The entire supernatant volume was filtered to concentrate cells and the filter cartridge was immediately frozen. A schematic of the experimental design can be found in Supplementary Figure 1.

### DNA/RNA extraction and sequencing

DNA and RNA were processed as previously reported and is detailed in the Supplementary Online Methods. Whole genome shotgun sequencing of the cathode-associated microbial community from the closed-circuit reactor (CCc) and the supernatant community from the closed circuit reactor (CCs) was accomplished with the Illumina MiSeq instrument yielding 250 bp paired-end reads totaling over 9 million reads with a total length of over 2 Gb for the attached microbial community, and over 5 million reads with a total length of over 1 Gb for the supernatant microbiome.

### Metagenome assembly and binning

Two metagenomes were assembled and annotated from the closed-circuit reactor (CCc and CCs). Near full-length 16S rRNA sequences from the CCc and CCs metagenomes were assembled over 100 iterations of the Expectation-Maximization Iterative Reconstruction of Genes from the Environment (EMIRGE) method^49^ following initial read mapping to a modified version of the dereplicated version of the SILVA 108 small-subunit (SSU) rRNA database^50^. During iterations, sequences 97% similar were clustered. Representative sequences were searched against the SSURef_111_NR_trunc database using BLASTN. Comparison of 16S rRNA, 16S rRNA gene, EMIRGE-assembled 16S rRNA gene, and metagenome-inferred taxonomy can be found in Supplementary Figure 2.

SPAdes^51^, Velvet+Metavelvet^52, 53^, Ray^54^, and IDBA-UD^55^ community genome assemblies were compared based on total length of the assembly, n50 score, and percent reads mapping back to the assemblies (Supplementary Table 1). Based on the assembly results, the SPAdes assembly was used to further analyze the metagenomes due to the highest percent of reads mapped to a threshold above 1 kb (cathode: 95.95%, supernatant: 91.61%) and total assembly size (cathode: 67,511,580 bp above 1 kb, supernatant: 42,119,276 bp). Following assembly, the SPAdes contigs were analyzed by Prodigal^56^ to predict protein-encoding genes. Prodigal-predicted amino acid sequences were then annotated using UBLAST searches (usearch64, September 2013^57^) against the uniref90 database^58^ with an e-value cutoff of 100. These annotations, along with kmer coverage and GC content were used for preliminary genome binning. Emergent self-organizing maps (ESOM) were used to refine the metagenome bins based on tetranucleotide frequency and contig coverage on contigs greater than 4 kb^59^. Smaller fragments (>1 kb) were then projected onto the 4 kb ESOM. To further refine the bins the multi-metagenome pipeline^60^ was used, leveraging differential genome coverages between CCc and CCs to aid contig identification. Results were compared to ESOM bins. Only contigs binned by both ESOM and multi-metagenome pipeline were used for reassemblies of individual genome bins. All reads associated with the contigs in each of the final bins were reassembled as single organism genomes by SPAdes using default options with the addition of the –careful flag and kmer sizes of 65, 77, 99, and 127. All contigs greater than 1 kb were uploaded to RAST^61^ for final annotation using RASTtk^62^. The re-assembled individual genome bins and one large “undetermined” bin were compiled into a combined metagenome for transcript mapping. See Supplementary Table 2 for bin summaries and completeness generated by CheckM^63^. The RASTtk annotations were compared to annotations^64^ from Pfam, COG, and a Blastx search of the non-redundant protein sequences database^65^. We used the HMMER web server and the Center for Biological Sequence Analysis tools for homology searches and predicting protein locations^66, 67^. The RAST-annotated protein encoding gene (peg) tags are used as identifiers throughout the manuscript with the bin abbreviation preceding the peg number. The annotations for the complete metagenome can be downloaded https://github.com/sirmicrobe/electrosynthesis.

### Metatranscriptome

Four total metatranscriptomes were recovered from CCc, CCs, OCc, and OCs. The FASTX toolkit was used to filter the reads with a quality score lower than 30 and a length less than 50 bp. Quality filtered reads from each sample were mapped to the CCc electrode metagenome contigs using BWA^68^. Raw hit counts mapped to each RAST-annotated protein encoding gene in the combined metagenome were then used to calculate reads per kilobase gene length per millions of mapped reads (RPKM) values. Number of metatranscriptome reads mapped per bin can be found in Supplementary Table 3. It should be noted that because of this approach of mapping to the CC metagenome, we could be missing transcript mapping to genes that have diverged through mutation from the time CC was inoculated into OC.

RPKM values for each gene were also normalized to bin coverage based on single copy *recA* gene abundances. This compensated for the abundance variations of the taxa between samples. Quantile normalization was used to evenly distribute the transcript values between sample conditions. Fold change for expression analysis between conditions was calculated by dividing RPKM/RecA-condition1 by RPKM/RecA-condition2. To eliminate 0 value errors and over interpretation of very low count genes, an empirically determined threshold value of 0.1 was added to all of the RPKM/RecA values. In addition to the differential gene expression between each reactor and location, we used the R package DESeq. 2 v1.16.1^69^ in R (v3.3.2) and treated the two cathodes as replicates and the two supernatant conditions as replicates in certain instances where they had similar transcript abundance despite the three hours at open circuit in the OC reactor. The R package ‘superheat’ v0.1.0 was used to generate the heatmaps.

### Metabolic model reconstruction

To construct models of our three highest relative abundance genomes (*Acetobacterium*, *Sulfurospirillum*, and *Desulfovibrio*), we imported the RAST-annotated versions of these genomes into the DOE Systems Biology Knowledgebase (KBase). Once in KBase, we used the *Build Metabolic Model* app, which is based on the ModelSEED algorithm^70^, to construct one draft model for each genome. Once the draft models were complete, we curated the models based on literature data, KEGG^71^, and MetaCyc^72^ (Supplementary Table 4c). This curation primarily involved the addition of electron utilization pathways (*Acetobacterium*), addition of electron transport chain reactions (*Sulfurospirillum*, and *Desulfovibrio*), and adjustment of reaction directionality to prevent pathways from proceeding in unphysiological directions.

All models were reconstructed, gapfilled, and curated in the KBase Narrative Interface, and the associated narratives include all parameters and media formulations used (https://narrative.kbase.us/narrative/ws.15248.obj.1). All model data is also available for download from the same narratives. More information on the model building can be found in the Supplementary Methods. The reactions for each model and condition can also be found in Supplementary Table 4a.

We carried out a comparative model analysis on thirty-three KEGG pathway categories involving gene presence and absence, transcriptomic data from CCc, flux analysis, and gap-filled reactions on each pathway category (Fig. 4). Methods for the prediction of metabolic activity and comparison to expression data can be found in the Supplementary Methods. Carbon fixation pathway categories (i.e. reductive TCA cycle and Wood- Ljungdahl pathways) were lumped into a single category called CO_2_ fixation. All of the metabolic models, their associated genomes, and the FBA analysis generated in this study are presented using the KBase Narrative Interface (NI) and are accessible at https://narrative.kbase.us/narrative/ws.15248.obj.1.

### Data availability

The metagenomic and metatranscriptome reads can be found in MG-RAST^73^ under the ID numbers (and names) 4673464.3–4673467.3 (ARPA_metagenome) for the metagenome (http://metagenomics.anl.gov/linkin.cgi?project=15936) and 4536830.3–4536839.3 (ARPA_metatranscriptome) for the metatranscriptomes (http://metagenomics.anl.gov/linkin.cgi?project=6065). Assembled contigs for *Acetobacterium*, *Sulfurospirillum*, and *Desulfovibrio* can be accessed at https://narrative.kbase.us/narrative/ws.15248.obj.1. Raw Illumina sequencing reads have been deposited in the NCBI SRA database under Bioproject PRJNA245339. Additionally, sequencing and assembly files can be found online at https://github.com/sirmicrobe/electrosynthesis.

## Electronic supplementary material

Supplementary Material

Supplementary Table 4
