# Supplementary material for: Metabolic Reconstruction and Modeling Microbial Electrosynthesis
Source: Sci Rep. 2017 Aug 21;7:8391. doi: 10.1038/s41598-017-08877-z (PMC5566340; doi:10.1038/s41598-017-08877-z)
Supplement: Supplementary file 1 — Supplementary Material [file 41598_2017_8877_MOESM1_ESM.doc]

**Metabolic Reconstruction and Modeling Microbial Electrosynthesis**

Christopher W. Marshall^1,3*^#, Daniel E. Ross^7^, Kim M. Handley^4*^, Pamela B. Weisenhorn^1^, Janaka N. Edirisinghe^4^, Christopher S. Henry^4^, Jack A. Gilbert^1,2,3^, Harold D. May^5,6^, and R. Sean Norman^7^#

1. Biosciences Division, Argonne National Laboratory, Argonne, Illinois, USA;

2. Department of Ecology and Evolution, The University of Chicago, Chicago, Illinois, USA;

3. Department of Surgery, The University of Chicago, Chicago, Illinois, USA;

4. Computing, Environment, and Life Sciences, Argonne National Laboratory, Illinois, USA;

5. Department of Microbiology and Immunology, Medical University of South Carolina, Charleston, South Carolina, USA;

6. Marine Biomedicine and Environmental Science Center, Medical University of South Carolina, Charleston, South Carolina, USA;

7. Department of Environmental Health Sciences, Arnold School of Public Health, University of South Carolina, Columbia, South Carolina, USA;

*Present address: Kim M. Handley, School of Biological Sciences, The University of Auckland, Auckland, New Zealand. Chris W. Marshall Department of Microbiology and Molecular Genetics, University of Pittsburgh

#Corresponding authors

Correspondence: CW Marshall, Department of Microbiology and Molecular Biology, University of Pittsburgh, 523 Bridgeside Point II, 450 Technology Dr., Pittsburgh, PA 15219. E-mail: [chris.w.marshall@gmail.com](mailto:chris.w.marshall@gmail.com). RS Norman, Department of Environmental Health Sciences, University of South Carolina, 931 Assembly St., Columbia, SC 29208. E-mail: rsnorman@sc.edu

**Contents**

**Supplementary Methods**

**Bioprospecting**

**Supplementary References**

**Supplementary Table 1. Assembly and mapping comparisons**

**Supplementary Table 2. Genome bin summary**

**Supplementary Table 3. Number of mapped metatranscriptome reads**

**Supplementary Figure 1. Experimental design**

**Supplementary Figure 2. Relative abundance of rRNA gene**

**Supplementary Figure 3. Volatile fatty acid production**

**Supplementary Figure 4. *Sulfurospirillum* TCA cycle genes**

**Supplementary Figure 5. SEM and EDX spectra**

**Supplementary Figure 6. Pathway flux and model agreement with transcripts**

**Attached: Supplementary Table 4. All reactions in the models and excretion profiles**

**See also:**

[**https://github.com/sirmicrobe/electrosynthesis**](https://github.com/sirmicrobe/electrosynthesis) **: spades_CCc_contigs_1kb.fa.zip : assembled contigs of CCc before binning, in fasta format**

[**https://github.com/sirmicrobe/electrosynthesis**](https://github.com/sirmicrobe/electrosynthesis) **: spades_CCs_contigs_1kb.fa.zip : assembled contigs of CCs before binning, in fasta format**

[**https://github.com/sirmicrobe/electrosynthesis**](https://github.com/sirmicrobe/electrosynthesis) **: binned_contigs.tgz : assembled contigs of each genome bin in fasta format**

[**https://github.com/sirmicrobe/electrosynthesis**](https://github.com/sirmicrobe/electrosynthesis) **: all_rast_pegs.tgz : all protein encoding genes (peg) sequences in fasta format**

[**https://github.com/sirmicrobe/electrosynthesis**](https://github.com/sirmicrobe/electrosynthesis) **: supplementary_table_gene_annotations.txt.gz : all annotated genes in the metagenome**

[**https://github.com/sirmicrobe/electrosynthesis**](https://github.com/sirmicrobe/electrosynthesis) **: all_annotated_rast_transcript_RPKM.csv.gz : all transcript data mapped to pegs**

**Supplementary Methods.**

**DNA/RNA extraction and sequencing**

Samples (frozen graphite granules or supernatant filters) were mixed with RLT Plus buffer (Qiagen), β-mercaptoethanol (10 μL per mL of RLT Plus), and DNase- and RNase-free silicon carbide beads (0.1 mm and 1 mm beads). Samples were subjected to five freeze/thaw cycles consisting of 1) snap freezing with liquid nitrogen until samples were completely frozen 2) incubation at 55^o^C until sample was completely thawed, and 3) intermittent vortexing for 2 minutes.

Nucleic acid (DNA/RNA) was extracted from lysed samples using an Allprep DNA/RNA Mini Kit (Qiagen), according to manufacturer’s instructions. RNA was purified further with an RNeasy kit (Qiagen). DNase treatment (TURBO DNA-free kit, ABI) was used to degrade residual DNA, and cDNA libraries were constructed from 100 ng of total RNA using random hexamers (SuperScript III, Life Technologies) according to manufacturer’s instructions. A MinElute PCR Purification Kit (Qiagen) was used to clean cDNA, which was used as a template for PCR amplification of the 16S rRNA gene (V3 region).

To generate cDNA of mRNA for transcriptome sequencing, messenger RNA (mRNA) was isolated from total RNA by removal of ribosomal RNA (rRNA) and tRNA using the Ribo-Zero kit (Epicentre), according to manufacturers instructions. Enriched mRNA was amplified using MessageAmp II-Bacteria Kit (Ambion) and converted to cDNA using SuperScript Double-Stranded cDNA synthesis kit (Invitrogen), according to manufacturers instructions.

We did amplicon sequencing of the 16S rRNA and rRNA gene of the supernatant and cathode communities in 4 reactors: CC, OC, CC2, and CC3. The pick_open_reference_otus.py script from QIIME version 1.7 was run using the uclust OTU picking method and the Greengenes database version 13.5 at 97% OTU cutoff to analyze the 16S rRNA amplicon sequencing data and following the standard pipeline described in the QIIME Illumina tutorial.^1^

**Metabolic model reconstruction, gapfilling, and curation**

Next, we applied the *Gapfill metabolic model* KBase app to identify and fill all the gaps in the metabolic pathways of our models that might prevent them from producing biomass while operating in one of our hypothesized metabolic roles within the microbial community. In this gap-filling process, the model was augmented to include all the reactions contained in the ModelSEED database (available for download from GitHub (https://github.com/ModelSEED/ModelSEEDDatabase/tree/master/Biochemistry). Additionally, all reactions determined to be thermodynamically reversible ^2–4^ were adjusted to be reversible. Finally, flux balance analysis (FBA) ^5^ was performed to generate a flux profile that produced biomass while minimizing the flux through all reactions and reaction directions that were not contained in the original model, consistent with previously published algorithms ^6,7^. All reactions and reaction directions not included in the original model that had a nonzero flux in were then added to the model as the gap-filling solution. This subsequently permits the growth of the gap-filled model in the specified condition. *Acetobacterium* was gapfilled in a condition that reflected the only proposed metabolic role for this organism: direct consumption of electrons to fix CO2 coupled to the production of acetate. The *Sulfurospirillum* and *Desulfovibrio* models were gapfilled separately in two distinct conditions reflecting the potential roles proposed for these organisms: (i) autotrophic growth on CO_2_ and H_2_; or (ii) heterotrophic growth on acetate. This produced a total of four models: (i) autotrophic *Sulfurospirillum*; (ii) heterotrophic *Sulfurospirillum*; (iii) autotrophic *Desulfovibrio*; and (iv) heterotrophic *Desulfovibrio*. The detailed composition of the electrosynthetic, autotrophic, and heterotrophic media formulations used in the gapfilling analysis are included in the supplementary material (Supplementary Table 4).

**Prediction of metabolic activity with comparison to expression data**

Flux balance analysis (FBA) was applied with each of our gapfilled metabolic models to simulate the production of biomass on the same media formulations used in our gapfilling analysis (electrosynthetic, autotrophic, and heterotrophic media). Thus, we simulated the growth of our gapfilled *Acetobacterium* model in our electrosynthetic media; we simulated the growth of our autotrophic *Sulfurospirillum* and *Desulfovibrio* models in our autotrophic media; and we simulated the growth of our heterotrophic *Sulfurospirillum* and *Desulfovibrio* models in our heterotrophic media (all media formulations are listed in 5b). We then maximized the production of biomass in each model, subject to the constraints on nutrient uptake imposed by our media formulations, and once a solution was produced, we minimized the sum of the fluxes comprising the solution, in order to produce a single solution that is both simple and distinct.

We validated our flux solutions by comparing the active reactions in each flux profile against the actively expressed genes identified based on our transcriptomic data. In order to conduct this comparison, we first needed to classify the genes in our three species as either *active* or *inactive* based on the relative abundance of mapped RNA-seq reads. This process began by identifying an initial set of universal genes in each species that can be assumed to be *always active* (e.g. DNA polymerase subunits and tRNA synthetases). These genes are listed in Supplemental table 4d. We ranked these *always active* genes based on the abundance of reads mapped to them, identifying the number of reads mapped to the gene ranked at the 10^th^ percentile in this list as the threshold expression value. Then in the broader genome, we labeled any gene with mapped reads that exceeded the threshold as active, with all other genes being inactive. Finally, we calculated the binary distance between the transcriptome and flux profiles, excluding any reactions that had been gapfilled, to determine which predicted flux profiles yielded the greatest agreement (i.e. lower binary distance) with the transcriptomic data.

**Scanning electron microscopy - energy dispersive x-ray spectroscopy (SEM-EDX)**

Cathodic graphite granules were fixed in 0.1 M sodium cacodylate buffer with 2% gluteraldehyde for three hours, washed in 2.5% osmium tetroxide, and then dehydrated with an ethanol dilution series using 0%, 25%, 50%, 75%, % and 100%.  Samples were stored in a desiccator and then coated with Au and Pd using a Denton Vacuum sputter coater.  Images were generated using a FEI Quanta 400 Scanning Electron Microscope.

**Supplementary Material**

**Bioprospecting.**

With the appropriate manipulation of the electrosynthetic microbiome, informed by the metagenome, metatranscriptome, and modeling predictions, other commodity chemicals could be produced from CO_2_. Based on this, a platform for chemical production is proposed that would be analogous to the carboxylate platform.^8^ This platform, called the Electrobiome® platform, would utilize a community of organisms to direct electrode-dependent CO_2_ fixation into a variety of different valuable chemicals. Since CO_2_ reduction to acetate by *Acetobacterium* is the foundation that the present community is built upon, the conversion of acetyl-CoA to other valuable products is of particular interest. Reduction of acetate to ethanol can be catalyzed by acetaldehyde dehydrogenase and alcohol dehydrogenases. *Sphaerochaeta*, *Acetobacterium*, *Sulfurospirillum*, *Desulfovibrio*, and *Geobacter* all have this capability according to each genome bin. Additionally, the metabolic model predicts the production of ethanol and propionate from *Acetobacterium*, and acetate, succinate, and malate from *Sulfurospirillum* (Supplementary Table 4). The Rhodobacteraceae, *Rhizobium*, and *Acetobacterium* bins all have complete acetyl-CoA to butyrate pathways, with the highest expression of this pathway by *Acetobacterium*. Furthermore, *Sulfurospirillum*, Bacteroidetes-h, *Sphaerochaeta*, Rhodobacteraceae, *Desulfovibrio*-l, and *Desulfovibrio*-h all have genes for butanol dehydrogenase. These genes indicate that given the right conditions, most of the acetate generated in the MES could be converted to butanol through versions of the well-studied *Clostridial*-type acetone-butanol-ethanol pathway.^9,10^

2,3-butanediol production from petroleum is a multi-billion dollar industry that is involved in a large variety of applications including synthetic rubber and solvents. Microbial production can replace petroleum using a pathway that converts pyruvate to acetoin with the acetolactate synthase and acetolactate decarboxylase before butanediol dehydrogenase converts acetoin to 2,3-butanediol. In the MES, only the *Acetobacterium* and *Sphaerochaeta* bins encode the acetolactate decarboxylase for acetoin production. However, Rhodobacteraceae, all *Desulfovibrio* bins, and *Rhizobium* are capable of converting acetoin to 2,3-butanediol with butanediol dehydrogenases. These organisms working together or combining the entire pathway into one of the community-compatible organisms could steer the production of CO_2_- derived 2,3-butanediol.

Another valuable product that could be an end-goal of microbial electrosynthesis is polyhydroxyalkonoates (PHAs).^11^ Analysis of the genomes of Rhodobacteraceae, *Acetobacterium*, *Bacteroidetes*-low, and *Rhizobium* reveal the presence of polyhydroxybutyrate biosynthesis pathways. Nitrogen or phosphorous starvation at the proper reactor phase could trigger these organisms to store carbon in PHAs that could be harvested and used for industrial applications such as biodegradable plastics.^12^

**References**

1. Caporaso, J. G. *et al.* QIIME allows analysis of high-throughput community sequencing data. *Nat. Methods* **7,** 335–336 (2010).

2. Henry, C. S., Jankowski, M. D., Broadbelt, L. J. & Hatzimanikatis, V. Genome-scale thermodynamic analysis of Escherichia coli metabolism. *Biophys. J.* **90,** 1453–1461 (2006).

3. Henry, C. S., Zinner, J. F., Cohoon, M. P. & Stevens, R. L. iBsu1103: a new genome-scale metabolic model of Bacillus subtilis based on SEED annotations. *Genome Biol.* **10,** R69 (2009).

4. Jankowski, M. D., Henry, C. S., Broadbelt, L. J. & Hatzimanikatis, V. Group contribution method for thermodynamic analysis of complex metabolic networks. *Biophys. J.* **95,** 1487–1499 (2008).

5. Orth, J. D., Thiele, I. & Palsson, B. Ø. What is flux balance analysis? *Nat. Biotechnol.* **28,** 245–248 (2010).

6. Dreyfuss, J. M. *et al.* Reconstruction and validation of a genome-scale metabolic model for the filamentous fungus Neurospora crassa using FARM. *PLoS Comput. Biol.* **9,** e1003126 (2013).

7. Latendresse, M. Efficiently gap-filling reaction networks. *BMC Bioinformatics* **15,** 225 (2014).

8. Agler, M. T., Wrenn, B. A., Zinder, S. H. & Angenent, L. T. Waste to bioproduct conversion with undefined mixed cultures: the carboxylate platform. *Trends Biotechnol.* **29,** 70–78 (2011).

9. Steinbusch, K. J. J., Hamelers, H. V. M., Schaap, J. D., Kampman, C. & Buisman, C. J. N. Bioelectrochemical Ethanol Production through Mediated Acetate Reduction by Mixed Cultures. *Environ. Sci. Technol.* **44,** 513–517 (2010).

10. Steinbusch, K. J. J., Arvaniti, E., Hamelers, H. V. M. & Buisman, C. J. N. Selective inhibition of methanogenesis to enhance ethanol and n-butyrate production through acetate reduction in mixed culture fermentation. *Bioresour. Technol.* **100,** 3261–3267 (2009).

11. Marshall, C. W., LaBelle, E. V. & May, H. D. Production of fuels and chemicals from waste by microbiomes. *Curr. Opin. Biotechnol.* **24,** 391–397 (2013).

12. Salehizadeh, H. & Van Loosdrecht, M. C. M. Production of polyhydroxyalkanoates by mixed culture: recent trends and biotechnological importance. *Biotechnol. Adv.* **22,** 261–279 (2004).

13. Kanehisa, M. & Goto, S. KEGG: Kyoto Encyclopedia of Genes and Genomes. *Nucleic Acids Res.* **28,** 27–30 (2000).

Supplementary Table 1. Comparison of different assemblers and mapping programs for analysis of the electrosynthesis metagenomes

| CCc: metagenomic reads | | | | | | | |
| --- | --- | --- | --- | --- | --- | --- | --- |
| **Assembler** | **Threshold (nucleotides)** | **Sum (nucleotides)** | **N50 (nucleotides)** | **bowtie2-2.1.0** | | **bwa-0.7.4** | |
|  |  |  |  | **mapped** | **paired** | **mapped** | **paired** |
| idba | 100 | 77795033 | 3027 | 92.11% | 65.36% | 97.57% | 90.23% |
| idba | 500 | 66343453 | 4728 | 88.74% | 63.80% | 95.41% | 90.27% |
| idba | 1000 | 54174065 | 7762 | 83.29% | 60.34% | 90.34% | 86.72% |
| idba | 2000 | 44373247 | 12551 | 75.35% | 54.86% | 82.65% | 80.03% |
| idba | 5000 | 32504340 | 25868 | 62.99% | 45.96% | 69.74% | 67.94% |
| meta-velvet | 100 | 41607972 | 44912 | 87.98% | 63.98% | 91.90% | 88.64% |
| meta-velvet | 500 | 40445369 | 46111 | 87.58% | 63.81% | 91.67% | 89.17% |
| meta-velvet | 1000 | 39407393 | 48544 | 86.74% | 63.24% | 91.19% | 89.22% |
| meta-velvet | 2000 | 37928752 | 51753 | 85.10% | 62.06% | 89.96% | 88.19% |
| meta-velvet | 5000 | 34935403 | 59963 | 82.19% | 59.91% | 87.37% | 85.73% |
| ray | 100 | 83702681 | 1608 | 93.26% | 67.84% | 97.35% | 93.52% |
| ray | 500 | 54343125 | 15724 | 90.81% | 66.42% | 94.23% | 92.20% |
| ray | 1000 | 46412963 | 22530 | 89.09% | 65.24% | 92.48% | 90.82% |
| ray | 2000 | 39955753 | 28883 | 86.91% | 63.66% | 90.42% | 88.95% |
| ray | 5000 | 34536327 | 35126 | 83.49% | 61.12% | 87.23% | 85.89% |
| spades | 100 | 102479092 | 3042 | 96.44% | 70.44% | 98.81% | 95.84% |
| spades | 500 | 85510647 | 6704 | 95.23% | 69.46% | 97.89% | 95.17% |
| spades | 1000 | 67511580 | 13120 | 92.78% | 67.74% | 95.95% | 93.70% |
| spades | 2000 | 55962799 | 19812 | 89.43% | 65.34% | 93.20% | 91.22% |
| spades | 5000 | 46079388 | 29877 | 83.28% | 60.90% | 88.07% | 86.26% |

| CCs: metagenomic reads | | | | | | | | |  |
| --- | --- | --- | --- | --- | --- | --- | --- | --- | --- |
| **Assembler** | **Threshold (nucleotides)** | **Sum (nucleotides)** | **N50 (nucleotides)** | **bowtie2-2.1.0** | | | **bwa-0.7.4** | | |
|  |  |  |  | **mapped** | | **paired** | **mapped** | **paired** |  |
| idba | 100 | 49997567 | 1902 | 88.43% | 46.37% | | 94.27% | 85.66% |  |
| idba | 500 | 41723580 | 2591 | 86.07% | 45.62% | | 91.40% | 86.31% |  |
| idba | 1000 | 33611698 | 3510 | 82.97% | 44.17% | | 88.02% | 84.05% |  |
| idba | 2000 | 24323949 | 5388 | 77.56% | 41.45% | | 82.40% | 79.38% |  |
| idba | 5000 | 12852473 | 11925 | 67.27% | 36.18% | | 71.49% | 69.38% |  |
| meta-velvet | 100 | 14756344 | 91737 | 80.76% | 43.24% | | 83.75% | 81.27% |  |
| meta-velvet | 500 | 14530692 | 93326 | 80.56% | 43.19% | | 83.55% | 81.45% |  |
| meta-velvet | 1000 | 14375123 | 93844 | 80.26% | 43.06% | | 83.37% | 81.48% |  |
| meta-velvet | 2000 | 14168259 | 95639 | 79.30% | 42.58% | | 82.65% | 80.96% |  |
| meta-velvet | 5000 | 13902647 | 96572 | 77.91% | 41.87% | | 81.42% | 79.89% |  |
| ray | 100 | 49073386 | 614 | 88.63% | 46.95% | | 93.43% | 86.87% |  |
| ray | 500 | 27185488 | 6477 | 85.13% | 45.66% | | 88.45% | 85.80% |  |
| ray | 1000 | 19029782 | 32496 | 82.42% | 44.30% | | 85.18% | 83.48% |  |
| ray | 2000 | 15047272 | 55474 | 80.68% | 43.43% | | 83.24% | 81.83% |  |
| ray | 5000 | 13964335 | 61149 | 79.56% | 42.82% | | 82.14% | 80.81% |  |
| spades | 100 | 66638920 | 1796 | 93.03% | 49.94% | | 95.24% | 91.30% |  |
| spades | 500 | 54751467 | 2752 | 91.45% | 48.90% | | 93.95% | 90.67% |  |
| spades | 1000 | 42119276 | 4484 | 88.83% | 47.48% | | 91.61% | 89.04% |  |
| spades | 2000 | 31680730 | 7549 | 85.15% | 45.56% | | 88.26% | 86.14% |  |
| spades | 5000 | 19815300 | 23189 | 79.61% | 42.69% | | 82.95% | 81.19% |  |

Supplementary Table 2. Summary of assembled genome bins.

| RAST ID | ESOM-TREE | EMIRGE | Relative abundance based on metagenome coverage | CCc average coverage | CCs average coverage | DNA (16S amplicon-seq) | RNA (16S amplicon-seq) | sequence size | number of contigs | longest contig | n50 | GC content | Completeness | Contamination |
| --- | --- | --- | --- | --- | --- | --- | --- | --- | --- | --- | --- | --- | --- | --- |
| Acetobacterium sp MES1 | Acetobacterium sp MES1 | 30.5559 | 21.25603865 | 88 | 18 | 25.5 | 22 | 4175392 | 711 | 251082 | 79566 | 44.5 | 100 | 12.91 |
| Sulfurospirillum sp MES13 | Sulfurospirillum sp MES9 | 23.9126 | 26.57004831 | 110 | 101 | 21.4 | 37.7 | 2641043 | 132 | 790048 | 727588 | 43.9 | 100 | 1.86 |
| Desulfovibrio high-cov MES4 | Desulfovibrio high-cov MES5 | 18.6082 | 21.98067633 | 91 | 3 | 14.8 | 19.3 | 3713194 | 367 | 299901 | 87645 | 57.6 | 100 | 5.16 |
| Geobacter sp MES8 | Geobacter sp MES3 | 1.658 | 2.657004831 | 11 | 0 | 0 | 0 | 3780232 | 285 | 368550 | 120623 | 57.1 | 99.42 | 5.32 |
| Sphaerochaeta sp MES12 | Sphaerochaeta sp MES10 | 1.6242 | 3.623188406 | 15 | 2 | 4.3 | 6.2 | 3659357 | 369 | 269385 | 112481 | 50.8 | 97.75 | 3.93 |
| Rhodobacteraceae sp MES11 | Rhodobacteraceae sp MES7 | 6.7809 | 5.314009662 | 22 | 9 | 8.4 | 4.5 | 7455206 | 5699 | 580519 | 87488 | 68.3 | 100 | 73.43 |
| Rhizobium sp MES10 | Rhizobium sp MES8 | 0.8557 | 1.207729469 | 5 | 2 | 1.5 | 0 | 4840830 | 1255 | 84020 | 12319 | 61.5 | 89.27 | 12.81 |
| Methanobacterium sp MES9 | Methanobacterium sp MES13 | 2.9 | 1.93236715 | 8 | 0 | n/a | n/a | 4077765 | 1419 | 43505 | 8808 | 40.9 | 98.15 | 110.09 |
| Desulfovibrio ultralow-cov MES7 | Desulfovibrio ultralow-cov MES4 | 0.0783 | 1.207729469 | 5 | 0 | n/a | 1.2 | 3191878 | 759 | 44321 | 9959 | 57.8 | 96.18 | 7.54 |
| Desulfovibrio low-cov MES6 | Desulfovibrio low-cov MES6 | 4.8359 | 4.106280193 | 17 | 2 | 4.2 | 1.9 | 4438010 | 776 | 277969 | 90453 | 66.1 | ? | ? |
| Desulfovibrio low-plasmid MES5 | Desulfovibrio low-plasmid MES6 | 0 | 0 | 5 | 2 | n/a | n/a | 2202288 | 762 | 22579 | 4903 | 58.6 | 64.72 | 9.17 |
| Bacteroid low-cov MES3 | Bacteroid low-cov MES12 | 1.8743 | 2.173913043 | 9 | 0 | 3.8 | 0 | 4230330 | 1397 | 128251 | 20712 | 44.1 | 94.83 | 34.58 |
| Bacteroid high-cov MES2 | Bacteroid high-cov MES11 | 3.058 | 7.246376812 | 30 | 2 | 5 | 0 | 4404814 | 316 | 216409 | 86325 | 46.9 | 97.15 | 4.86 |
| undetermined | undetermined | 3.258 | 0.724637681 | 3 | 3 | 7.2 | 7.2 | 32358418 | 27303 | 87911 | 1489 | 48.9 | N/A | N/A |

Supplementary Table 3. Number of metatranscriptome reads mapped to each genome bin.

| Genome Bin | CCc | OCc | CCs | OCs |
| --- | --- | --- | --- | --- |
| sulfuro-MES9 | 5014847 | 1329057 | 20444562 | 2084854 |
| dsv-h-MES5 | 269362 | 313154 | 116392 | 225512 |
| aceto-MES1 | 1945728 | 1217609 | 275205 | 489151 |
| bacteroid-h | 877811 | 1845845 | 51148 | 317327 |
| geo-MES3 | 34347 | 16616 | 2897 | 7720 |
| rhodo-MES7 | 131987 | 329044 | 101882 | 261643 |
| dsv-l-MES6 | 38440 | 27302 | 23780 | 22592 |
| dsv-l-p-MES6 | 18998 | 52412 | 32222 | 48207 |
| dsv-ul-MES4 | 22020 | 47381 | 46028 | 59066 |
| sphaer-MES10 | 162054 | 212824 | 55031 | 78157 |
| methan-MES13 | 76078 | 131361 | 4513 | 40815 |
| rhizo-MES8 | 47961 | 116307 | 25133 | 78155 |
| bacteroid-l-MES12 | 421225 | 705760 | 78266 | 115282 |
| undetermined | 5804642 | 5960223 | 2984571 | 8691478 |
| total | 14865500 | 12304895 | 24241630 | 12519959 |

Supplementary Figure 1. Diagram showing the overall experimental design and structure of MECs.

Supplementary Figure 2. Relative abundance of the 16S rRNA and rRNA gene amplicons (Bacteria-specific primers) in each reactor and location (A), relative abundance of community members based on metagenome coverage and EMIRGE near full length 16S rRNA gene calculations (B), and relative abundance based on metatranscriptomic reads mapping to different conserved marker genes found in the metagenome (C).

A. B.

C.


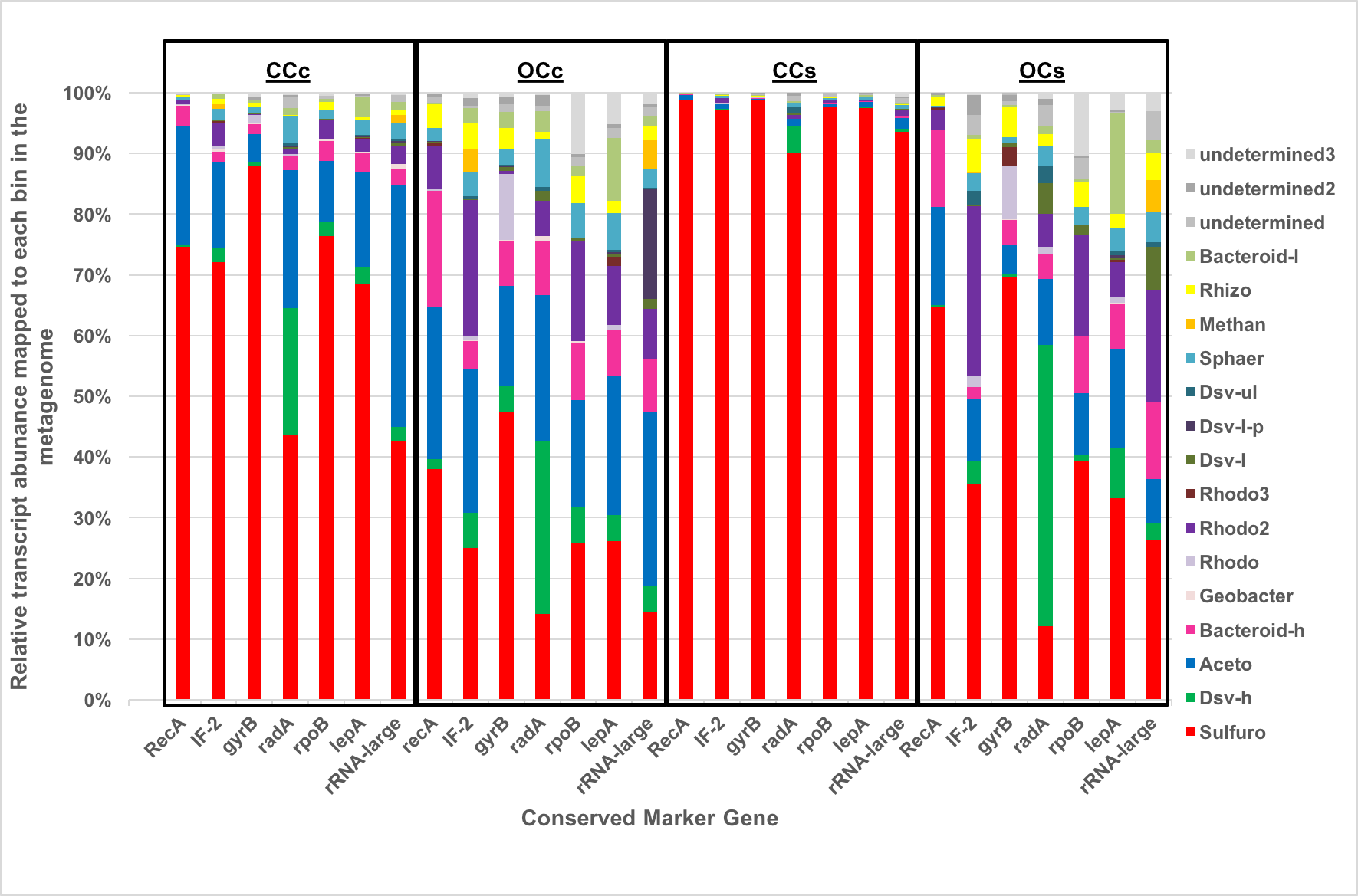


Supplementary Figure 3. Volatile Fatty Acid (VFA) production in CC (A) and OC (B) reactors over select time periods with relatively high VFA production.

**A.**


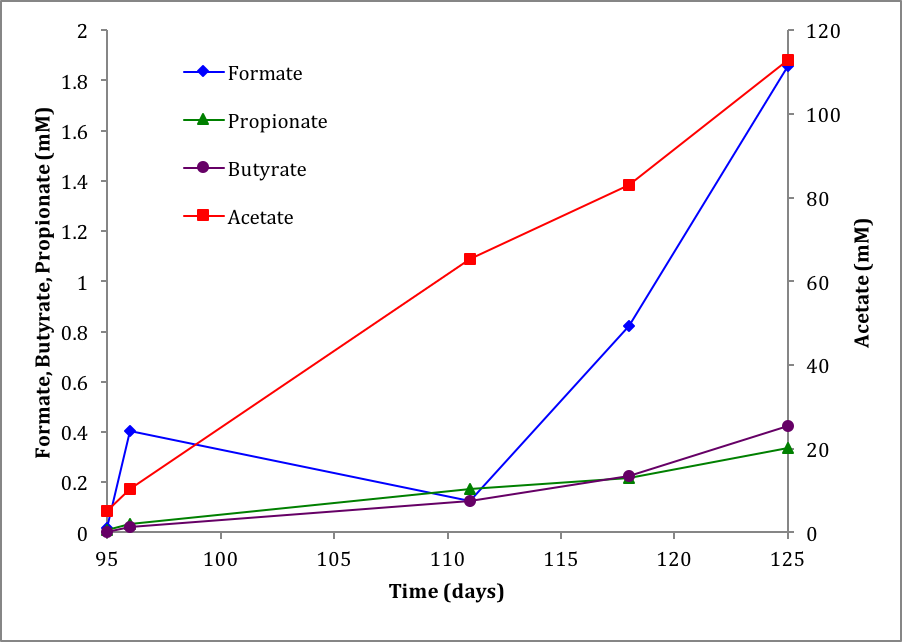


**B.**


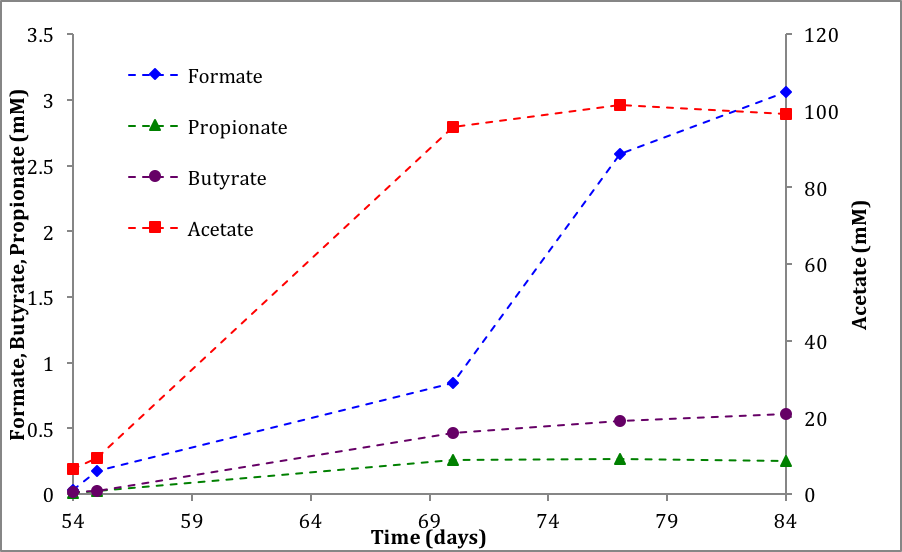


Supplementary Figure 4. Expression of rTCA cycle genes in Sulfurospirillum. (A) relative expression of TCA and reverse TCA cycle components and (B) confirmed rTCA components shaded in green on KEGG map.^13^

**A. B.**

Supplementary Figure 5. SEM images and EDX spectra of (A) electrode-attached cells, (B) extracellular component, and (C) uninoculated electrode.

**A. B. C.**


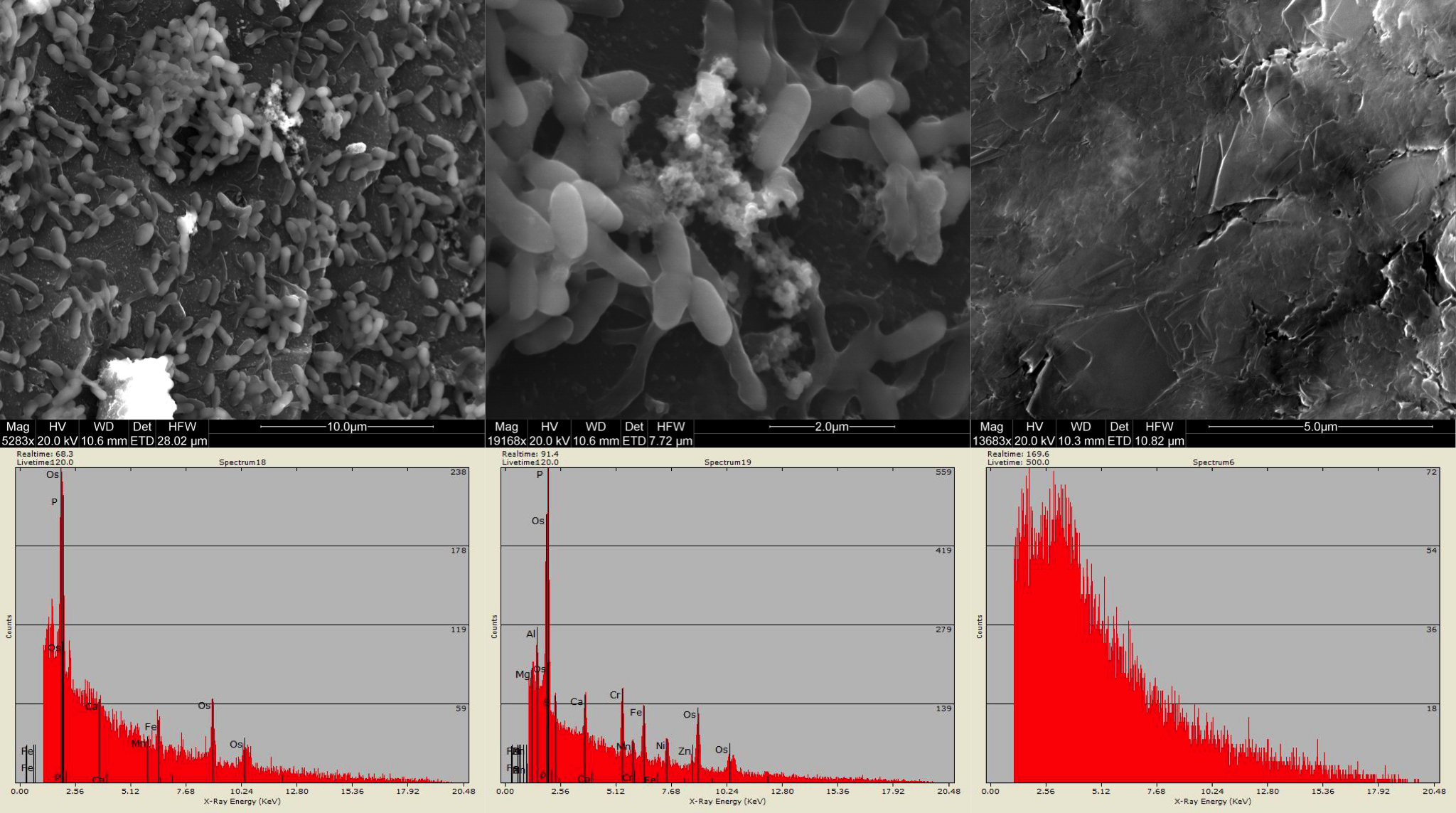


Supplementary Figure 6. KEGG pathway flux and model agreement with expression data of the three predominant members of the microbial community.


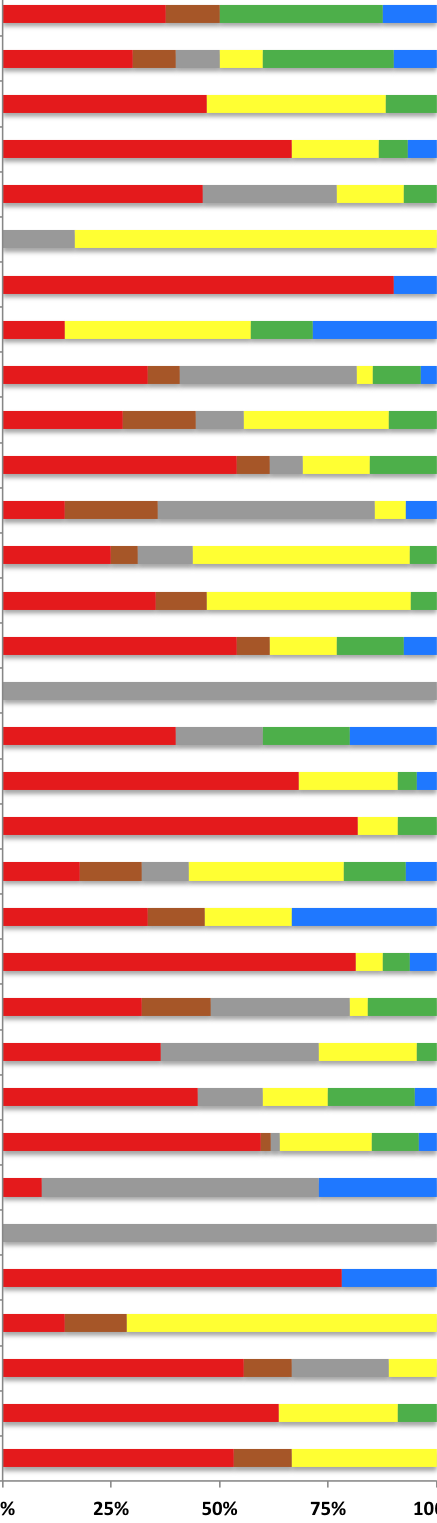

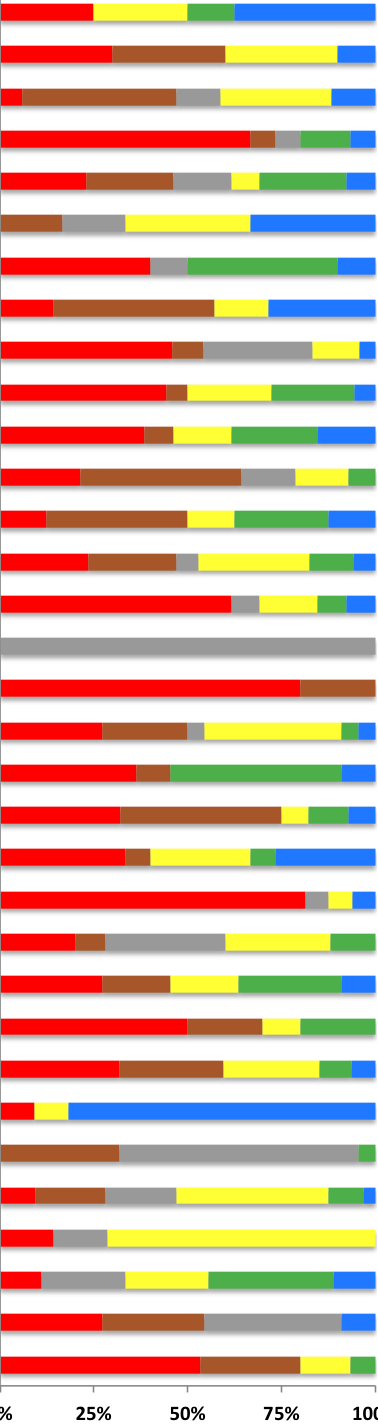

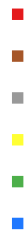

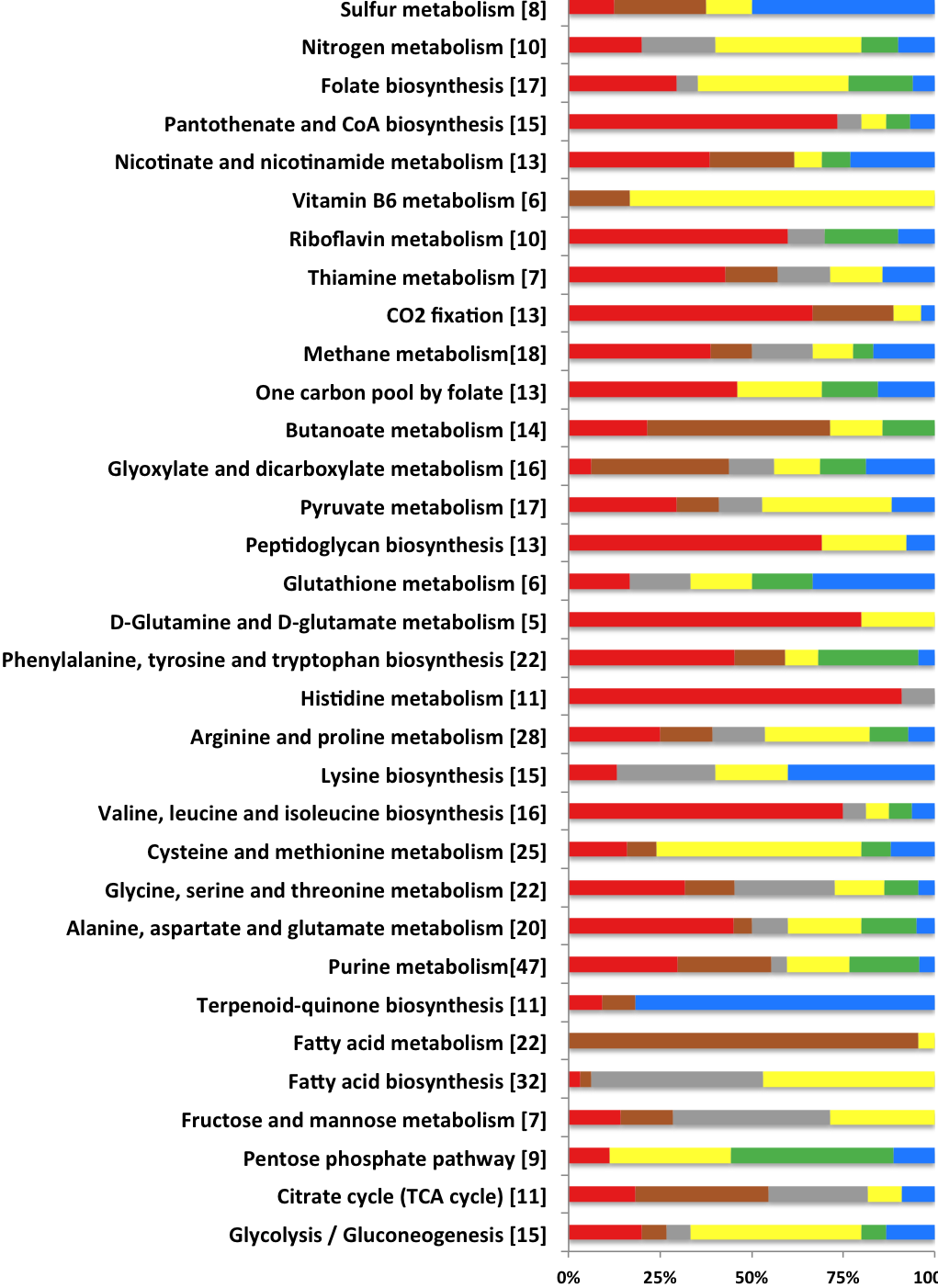


**Acetobacterium Sulfurospirillum Desulfovibrio**

GSA reactions F(+) E (+)

GSA reactions F(-) E (-)

GSA reactions F(+) E (-)

GSA reactions F(-) E (+)

Gapfilled reactions F(+)

Reaction not in models

GSA – Gene Associated Reactions

F - Metabolic Reaction Flux

E – Expression
